# A conceptual framework for automation disengagements

**DOI:** 10.1038/s41598-024-57882-6

**Published:** 2024-04-15

**Authors:** S. Nordhoff

**Affiliations:** Department Transport and Planning, Delft University of Technology, Delft, The Netherlands

**Keywords:** Partial driving automation, Automation disengagements, Disuse, Human factors, Tesla’s Full Self-Driving (FSD) Beta, Human behaviour, Civil engineering

## Abstract

A better understanding of automation disengagements can lead to improved safety and efficiency of automated systems. This study investigates the factors contributing to automation disengagements initiated by human operators and the automation itself by analyzing semi-structured interviews with 103 users of Tesla’s Autopilot and FSD Beta. The factors leading to automation disengagements are represented by categories. In total, we identified five main categories, and thirty-five subcategories. The main categories include human operator states (5), human operator’s perception of the automation (17), human operator’s perception of other humans (3), the automation’s perception of the human operator (3), and the automation incapability in the environment (7). Human operators disengaged the automation when they anticipated failure, observed unnatural or unwanted automation behavior (e.g., erratic steering, running red lights), or believed the automation is not capable to operate safely in certain environments (e.g., inclement weather, non-standard roads). Negative experiences of human operators, such as frustration, unsafe feelings, and distrust represent some of the adverse human operate states leading to automation disengagements initiated by human operators. The automation, in turn, monitored human operators and disengaged itself if it detected insufficient vigilance or speed rule violations by human operators. Moreover, human operators can be influenced by the reactions of passengers and other road users, leading them to disengage the automation if they sensed discomfort, anger, or embarrassment due to the automation’s actions. The results of the analysis are synthesized into a conceptual framework for automation disengagements, borrowing ideas from the human factor's literature and control theory. This research offers insights into the factors contributing to automation disengagements, and highlights not only the concerns of human operators but also the social aspects of this phenomenon. The findings provide information on potential edge cases of automated vehicle technology, which may help to enhance the safety and efficiency of such systems.

## Introduction

Since October 2020, drivers in the United States and Canada have been using Tesla’s Full Self-Driving (FSD) Beta feature, a partial (or SAE Level 2) automated driving system that expands the operational design domain of Autopilot beyond highways to non-highway roads. Despite the name “Full Self-Driving”, drivers must constantly monitor FSD Beta and intervene, as it may malfunction at inopportune moments. Drivers were instructed by Tesla via email prior to their first use to keep their hands on the wheel, pay extra attention on the road, and avoid complacency^1^.

The study of human misuse and disuse of automation has been a subject of research since the 1990s. Misuse has typically been associated with overtrust, while disuse has been associated with distrust in the automation^2,3^. Automation misuse refers to an inappropriate reliance on the automation’s capabilities^4,5^. Overreliance can impact human operator’s decision (whether conscious or sub-conscious) to become complacent with the automation^2^. Research indicates that some Tesla Autopilot users misused the automation, becoming complacent and performing hazardous behaviors such as hands-free driving and mind-off driving, intentionally manipulating the steering wheel to feign attentiveness, or sleeping while the system was engaged^1,6–8^. Teoh^9^ showed that the name “Autopilot” was associated with the highest likelihood of respondents believing that engaging in secondary tasks (e.g., talking with passengers, hands off the steering wheel, foot not near the pedals) is safe while in operation in comparison to the names of four other commercially available Level 2 systems. Recently, ^10^ documented both an objective and self-reported increase over time in secondary task engagement when using Level 2 automated driving systems. Respondents subjectively reported an increased trust in the automated driving system, and an increased focus on the road with the automation. These findings correspond with other studies documenting an increase in trust in certain advanced driver assistance systems (ADAS) over time^11–13^.

The term ‘disuse’ refers to the underutilization of automation^2^, and is commonly applied to situations in which human operators decide not to use the automation (i.e., not turning on, or turning the automation off after usage), leading to the rejection of the capabilities of the automation^2^.

This current article examines automation disengagements, which are defined as the deactivation of the automated driving system by human operators or the automation itself^14^. This is different from automation disuse, which may manifest in a more enduring manner. Research on the disengagements of automated driving systems has demonstrated that human operator-initiated disengagements occurred in anticipation of potentially hazardous situations, such as adverse weather conditions, construction zones, poor road infrastructure, the presence of emergency vehicles, or navigating curves. Other reasons for human operators to disengage the automation were to execute lane-changing maneuvers, lack of trust or discomfort, testing the limits of the automation, overly cautious behavior of the automation, other road users’ (reckless) driving behavior, or for no specific reasons^14–21^. Adverse human operator states, such as time pressure, boredom, fatigue, anger or frustration, motivated human operators of vehicles equipped with automated driving systems to disengage the automation^19^. In turn, automation-initiated disengagements occurred in response to system failure or missing functionality^19,22^, such as failures in sensor and detection technology, communication systems, map calibration, or hardware^15^. An extensive analysis of 14,525 disengagements over a six-year period has shown that discrepancies pertaining to estimating the location and trajectory of the vehicle, weather, road conditions, and the driving environment, as well as hardware and software were the first, second, and third main causes underlying the disengagements, respectively^23^.

The safety benefits of partial driving automation beyond crash avoidance remain unclear^24^. On the one hand, automation disengagements may pose a safety concern because they can result from the automation exceeding the limits of its operational design domain, requiring human operators to reclaim control within short time margins. Conversely, it is possible that partially automated driving systems help to prevent situations in which crash avoidance systems would be activated^24^. Understanding the reasons for disengaging partial driving automation is essential to obtain knowledge about the situations exceeding the capability of human operators to use the automation as intended. Knowledge about disengaging the automation can also provide valuable insights into different ‘edge case’ scenarios, namely situations in which human operators disengage the partially automated driving system because its operational boundaries have been exceeded. Addressing these edge cases is pivotal to fully realizing the benefits of partial driving automation and minimizing its risks^25^, contributing to its widespread acceptance.

### The present study

The present study examines the disengagement of partial driving automation, which can either be human operator- or automation-initiated. There is a scarcity of research on the factors underlying human operator- and automation-initiated disengagements of Autopilot and FSD Beta. Previous studies have examined various factors contributing to disengagements, such as the impact of automation capability on trust and reliance, as well as the influence of operator confidence, risk perception, and learning. Most of the research reporting disengagements of partially automated driving systems has relied on technical vehicle data^16,26,27^ rather than in-depth qualitative accounts from the users of these systems. Obtaining knowledge on the psychological aspects of disengaging Autopilot and FSD Beta enhances our understanding of the factors contributing to automation disengagements^27^. This study adopts a comprehensive approach by examining the reasons behind the disengagements of complex real-life partially automated driving systems: Tesla Autopilot and FSD Beta.

## Method

### Recruitment

We conducted semi-structured interviews with users of vehicles equipped with Tesla’s partially automated driving systems ‘Autopilot’ and ‘FSD Beta’. The study was approved by the Human Research Ethics Committee (HREC) of Delft University of Technology (ID: 2316). The study was performed in accordance with relevant guidelines and regulations as mentioned in the documents submitted to the HREC for study approval. These documents consisted of a risk assessment and mitigation, and data management plan, as well as written informed consent form submitted to respondents prior to study participation to inform them about the nature and purpose of the research.

We recruited respondents through specialized online communities and forums (i.e., Discord, Facebook, Twitter, Reddit, YouTube, Instagram, Tesla Motors Club, and Tesla Motors Forum). As FSD Beta was only available to in North America and Canada during the study, we mainly recruited in these regions. Ownership of a Tesla was subjectively evaluated using self-reported data regarding access to Autopilot and FSD Beta.

### Procedure

The interviews were conducted online via Zoom, with both audio and visual recorded. The interviews followed a pre-defined protocol consisting of open-ended and closed-ended questions. To reduce the subjectivity of interview research (interviewer bias) and ensure consistency in the implementation of the interviews across respondents, several steps were conducted as outlined below.

An interview protocol was created on Qualtrics (https://www.qualtrics.com/de/), and a link to the questions was sent through the chat function of Zoom at the beginning of the interview. This allowed respondents to view the questions directly, allowing them to advance to the next question themselves. Respondents were allowed to skip questions that were already answered by previous questions. The interviewer listened closely to respondents as they navigated through the questionnaire to reduce the risk of influencing respondents during the interview. However, in line with semi-structured qualitative interview research^28^, the interviewer asked follow-up questions to clarify aspects raised by respondents, explore new phenomena that were not covered by the original interview protocol, or to provide assistance in providing definitions of study constructs when requested by respondents or when the interviewer had the impression that assistance would be needed. As the questions were standardized and logically ordered, the researcher’s intervention was limited.

Our method was specifically designed to mitigate the subjectivity associated with interviewer-respondent interaction. By sharing the questions with respondents beforehand, we reduced potential bias from the tone of the interviewer or question wording, as “certain words hold power and can affect how respondents ‘hear’ the question”^29^, p. 43. Furthermore, by allowing respondents to view the questions in front of them, any embarrassment or frustration that could arise by respondents not understanding the meaning of the questions could be reduced^29^.

The interview protocol comprised two main parts. Initially, respondents provided their written informed consent to participate in the study. The first part consisted mostly of open-ended questions, while the second part primarily involved closed-ended questions about respondents’ socio-demographic profile, travel behavior (e.g., age, gender, education, frequency of Autopilot and FSD Beta use), and general attitudes towards traffic safety.

Table A1 in the appendix presents an overview of the questions asked in the first part of the interview. With the focus on automation disengagements, the present study only addressed the questions Q28, “Do you disengage Autopilot and FSD Beta? Why / why not?”, and Q29, “Does Autopilot and FSD Beta disengage? When / in which situations?” Respondents were asked to answer each question separately for Autopilot and FSD Beta, reflecting on any differences between the systems. The questions Q1, Q4, Q24*–*Q26, and Q30*–*Q35 were addressed in our previous study^1^.

### Data analysis

The data analysis was performed in five steps:

1. Interviews were recorded via Zoom and transcribed using Microsoft Teams transcription software. Transcripts were compared with audio files and corrected as necessary.
2. A content analysis was conducted to inform the development of categories and subcategories^30^. These categories were derived deductively and inductively from the data. To inform the deductive category development, the categorization scheme of the disengagement causes from^14^ was used, which classifies disengagements in terms of control discrepancies, environmental and other road users, hardware, software, perception, and planning discrepancies, and operator takeover. Subcategories that did not correspond with this classification scheme were developed inductively by applying common text analysis methods, such as writing notes, searching for keywords, and jumping between text passages. The development of subcategories was based on repetitions, similarities and differences of key words and phrases. Categories were clearly defined to be operationalized in future studies, and understandable to experts and laymen. The development of the categories was an emergent and iterative process. This part of the analysis was conducted in Atlas.ti (Version 22.0.2). To qualify as subcategory, it had to be mentioned by at least five respondents.
3. To develop a conceptual framework, which links the categories identified in the previous step, the grounded theory approach suited for this purpose was used^31,32^. This step consisted of synthesizing the categories into the conceptual framework and was deemed to be completed “until the researcher recognizes a theoretical framework that makes sense”^32^, p. 54. In line with^31^, our framework enables the explanation and prediction of automation disengagements. The categories representing the reasons for disengagements are the predictor variables in our conceptual framework, while the disengagement itself represents the outcome variable. These categories were linked in this framework, allowing the derivation of hypotheses about the relationships between these categories in future research. Our framework also borrows ideas from control theory and the human factors literature describing the transitions of control as switch between automated and manual control in a model-based fashion^18,33–35^.
4. The occurrence of the subcategories was counted using seed terms or key terms developed inductively from the data, and deductively from the literature, which reflects a common practice^36^. An overview of the seed terms is provided in Table A2 in the appendix. Prior to the identification of the occurrence of the subcategories, the text was preprocessed and cleaned: Words (or tokens) with $\le$ 2 and $\ge$ 30 letters, and digits, hashtags, or hyperlinks, were removed. Next, words were transformed to lowercase, and stop words and words that did not add any substantial meaning to the sentences were removed. To count the occurrence of the subcategories, it was assumed that each subcategory represented by two seed terms (i.e., one seed term representing the reason for disengagement, and one seed term representing the disengagement itself) should occur in a window size of 10. This means that the occurrence of the subcategories was looked for in the 10 words before and 10 words after the seed terms, respectively. A subcategory was assigned a frequency of 1 if at least two seed terms were mentioned in this defined window size. The total number of mentions of a subcategory equals the total number of occurrences of at least two seed terms capturing the subcategory in all sentences of the interview transcripts. This analysis was conducted using Python.
5. The meaning of each subcategory was documented by quotes. Multiple mentions of a subcategory by respondents were not discarded but combined with other mentions of the subcategory by the respondent. As a result, some quotes represent collections of sentences mentioned by the same respondent at different points during the interview. A maximum of three quotes was selected to represent each subcategory.

## Results

The majority of respondents was male (91%), with an average age of 42 years, highly educated (52% had a Bachelor or Master degree), occupying positions as engineers (30%), or managers (8%), or were retired (7%). They predominantly resided in California (20%), Colorado (8%), and Florida (7%). Eighty-two percent of respondents utilized both Autopilot and FSD Beta, while 18% reported having access only to Autopilot.

The content analysis of the interview data led to the identification of the reasons for the human operator- and automation-initiated disengagements as presented by Table 1. The reasons for the disengagements were divided into five main categories, and thirty-five subcategories. The main categories include human operator states (5), human operator’s perception of the automation (17), human operator’s perception of other humans (3), the automation’s perception of the human operator (3), and the automation incapability in the environment (7). The results presented in Table 1 will be discussed in the subsequent sections.

**Table 1:** Factors contributing to human operator- and automation-initiated disengagements (main category, subcategory, meaning, type of disengagement (i.e., human operator, automation), type of system (i.e., Autopilot, FSD Beta), *n* (number of occurrences of disengagement factor).

Main category	Subcategory	Meaning	Type of disengagement		Type of system		*n*
			Human operator-initiated	Automation-initiated	Autopilot	FSD Beta	
Human operator states							
	Fatigue and intoxication	Fatigue and intoxication	X			X	7
	Frustration and stress	Frustration and stress associated with behavior of automation	X			X	17
	Embarrassment	Feeling of embarrassment towards other road users due to behavior of automation	X			X	2
	Travel trip constraints	Travel time, distance, and purpose constraints, with human operators initiating disengagement due to travel time constraints (in a hurry), distance constraints (distance too short), or trip constraints on the first and last end of trip, with automation not being able to initiate parking	X		X	X	14
	Enjoyment of manual driving	Enjoyment of manual driving	X		X	X	17
Human operator’s perception of automation							
	Software releases	New software releases and bugs associated with software release, with human operators engaging in short-term usage of automation to test and experience its limits or temporarily stop using it	X			X	35
	(Anticipated) automation failure	To avoid potential collision before automation can perform maneuver in situations in which operator capability in own skills exceeds perceived system capabilities to handle situation safely due to feelings of operator discomfort, low perceived safety, and lack of trust, contributing to frequent disengagements	X			X	138
	Unnatural / non-human-like automation behavior	To correct for unnatural and non-human-like automation behavior	X			X	22
	Random disengagements	Random disengagements without human operators knowing reasons underlying disengagements		X	X	X	8
	False positives	Disengagements despite human operators supervising automation		X	X		6
	Unwanted action(s)						
	Operational						
	Harsh deceleration	Harsh and sudden deceleration, commonly known as ‘phantom braking’, associated with shift of Tesla’s sensor suite from radar to vision-only, potentially increasing incidences of rear-end collisions and contributing to temporary disuse of automation		X	X	X	25
	Erratic steering wheel movements	Erratic, jerky steering wheel movements of automation	X			X	18
	Steering into adjacent traffic	Steering into adjacent traffic	X			X	11
	Tactical						
	Undesired lane changes	Unexpected, unnecessary, aggressive, or conservative lane changes	X		X	X	21
	Misidentifying correct lane	Steering into parking, bike, or bus lane	X			X	2
	Creeping	Creeping at intersections and in turning situations	X			X	6
	Rolling through stop signs	Rolling through stop signs, representing an illegal yet accepted human behavior	X	X		X	6
	Running red lights / stop signs	Running red lights and stop signs	X	X	X	X	3
	Unprotected left turns	Turning left at intersection with a green light instead of green arrow with oncoming traffic having the right of way	X			X	12
	Other turning situations	Other turning situations, such as ‘turning-on-red’, protected left-hand and right-hand and U-turns	X			X	186
	Strategic						
	Route unfamiliarity	On unfamiliar roads due to human operator’s lack of trust in automation given lack of experience in these areas	X		X	X	2
	Taking the wrong route	Taking wrong route, possibly because of misalignment between mapping and navigation data	X		X	X	15
Human operator’s perception of other humans							
	Discomfort	Passenger discomfort with perceived lack of control, or unpredictable and erratic system behavior	X		X	X	28
	Road rage	To avoid confusion and rage of other road users	X			X	12
	Reckless behavior	To respond to reckless driving behavior of other human operators, following too close from behind, or swerving into vehicle’s lane	X			X	14
Automation’s perception of the human operator							
	Complacency	Human operators failing to monitor automation		X	X		19
	Inappropriate amount of torque applied to the steering wheel	Human operators accidentally disengaging automation by applying inappropriate amount of torque to steering wheel	X	X	X		10
	Speed rule violation	Human operators exceeding speed limit for reasons of safety, efficiency, lack of knowledge, or complacency	X	X	X	X	8
Automation incapability in environment							
Weather	Inclement weather conditions	Inclement weather and poor visibility conditions	X	X	X	X	75
Road infrastructure and design	Non-standard roads	On roads with inconsistent and missing lane markings and with non-standard lane width	X	X	X	X	10
	Curves, hills	In curves and on hills	X	X	X	X	32
	Objects and events	Responding to stationary (e.g., potholes, road debris, speed bumps, bushes / trees, buildings, parked cars, construction, railway crossings, gates), and non-stationary objects (e.g., vulnerable road users, emergency vehicles, and other vehicles) in environment due to automation not being capable of identifying and responding to objects, with human operators consequently swerving around them, or lane-centering of system	X	X	X	X	149
	Intersections	In intersections, including roundabouts	X	X	X	X	62
	Discontinuities in road design	On-ramp and off-ramp, lane merging, and splitting situations	X	X	X		87
	Complex, heavy traffic	In heavy, complex traffic situations	X			X	80

### Human operator states

Human operators disengaged the automation due to adverse emotional states, such as fatigue, intoxication, frustration, or stress, because they faced travel trip constraints, or preferred manual driving.

#### Fatigue and intoxication

This subcategory covers human operator-initiated disengagements resulting from human operators being physically or mentally impaired by fatigue and intoxication.

“I didn’t use it on that trip because I was tired, and Beta is one of those things you have to be on top of it. You can’t use it when you’re impaired in any way, when you’re super tired, when you’ve had any alcohol.” (R096)

#### Frustration and stress

This subcategory covers human operator-initiated disengagements resulting from human operator’s frustration and stress with the behavior of FSD Beta.

“FSD Beta ‒ it’s less stressful to not have it enabled.” (R003)

“The only thing that impacts whether or not I use it is ‘Do I want to not have to worry about driving on a freeway?’ I’ll use Autopilot if I don’t really care. FSD Beta ‒ it’s not an unsafe thing that stops me from using it. It’s annoying to use it. I don’t want to babysit the car, so I’d rather just drive myself.” (R055)

“FSD Beta ‒ I just disengage it because at some point it becomes dangerous, and it’s easier for me. I get frustrated. Like ‘What are you? Why? Why are you doing this?’ ‘What’s the point of doing this?’ It’s just unnecessary.” (R058)

#### Embarrassment

This subcategory addresses human operator-initiated disengagements resulting from human operator’s feeling of embarrassment towards other road users due to the behavior of FSD Beta.

“I feel shame and embarrassment because it’s like ‘Oh, if I turned on my blinker and they gave me room, but I didn’t actually need to change lanes.’ Now they are confused, and I feel bad because I’m the worst driver on the road. I wanted a bumper sticker like ‘It’s not my fault. I’m so sorry. It’s a student driver.’” (R007)

“A woman and her dog standing on the corner of the road and I thought ‘I’m gonna turn off Full Self-Driving because Beta might turn really stupidly, and then this woman’s gonna be like ‘Wow, why does she make such a horrible turn?’ Would be embarrassed.” (R059)

“When it came up into that intersection, it just literally jerks you into the next lane with no notice, no reason, and that’s the scary thing. Especially if there was somebody there, it would be like ‘Was this guy drunk? Is he texting?’” (R069)

#### Travel trip constraints

Human operators reported to disengage FSD Beta when they faced travel time constraints, preferred to travel short distances, and at the beginning and end of a trip.

“When I take my lunch, it’s sort of ‘OK, I’m just gonna rush really quick right around the corner, grab a sandwich, and then rush right back to my apartment’, and it’s such a short distance that it doesn’t really warrant using it. So, that would be about the only time that I don’t use it.” (R049)

“I usually can’t engage Beta from my driveway. I can’t tell it ‘Leave my driveway’ or ‘Back into my drive’. So, once I get on my main road, I will usually engage it.” (R071)

“I turn off FSD Beta when I have to make a tight turn in and out of parking lots. The camera system is limited in what it can see, and it will nose out and then stop, or it will nose out and keep going, and you don’t want it to.” (R099)

#### Enjoy manual driving

The preference for manual control to enjoy driving contributed to human operator-initiated disengagements.

“I will disengage Autopilot if I want to have a little bit of fun. The car will never use its full acceleration capability when it’s in Autopilot or Full Self-Driving, and so if I’m the lead person at a stoplight, and there’s nobody that’s going to run a red, I’ll turn off Autopilot so that I can be more aggressive.” (R037)

“Almost the only time I ever turn it off is if I’m doing a joyride, or in the mountains, and really wanna experience the driving experience.” (R065)

### Human operator’s perception of automation

In addition to disengaging the automation due to these adverse emotional states or constraints, human operators initiated the disengagement due to new software releases, in anticipation of automation failures in environments exceeding the capabilities of the automation, or when the automation performed unwanted strategical, operational, and tactical driving maneuvers.

#### Software releases

This subcategory addresses human operator-initiated disengagements caused by new software releases, with human operators reporting to engage FSD Beta after a new software update has been released to test and experience the limits of the system, or temporarily disengaged FSD Beta due to software bugs associated with the software release.

“With each update, I like to test the new update on the same sections of road, and if it does something really bad a few times, I won’t use it for that whole update, but then I’ll try it again in the next update, and it might improve.” (R043)

“If FSD Beta is just a bad release, I just stopped using it most of the time because it’s pretty much unsafe.” (R078)

“The only way that would make me not to want to use FSD is that if I had a buggy software that they released.” (R091)

#### Anticipated automation failure

This subcategory addresses human operator-initiated disengagements in anticipation of upcoming system failure, with human operators reporting a high number of human operator-initiated disengagements.

“There is some point where we were going to lunch, my programmer and I, and I took it the two miles into town. It was so bad it would disengage every quarter mile, which is terrible.” (R067)

Human operators reported to taking over control before the car executed the maneuver because of discomfort, a lack of trust in the system’s capabilities, and low perceived safety. Some of these disengagements might have been unnecessary as it is not clear whether the system would have caused a crash if human operators had not intervened.

“You immediately have to intervene now. You don’t know if it would have turned itself because you intervened. I mean, even Tom was riding with me at that point. I was driving, and he said ‘Yeah, it would have turned, but it looked scary.’” (R067)

“I think to myself ‘Holy shit, if I didn’t take over in less than a half second, I am going to have a head-on collision with another car. I think that it would have corrected itself but then at the same time it’s the repercussions, if it didn’t correct itself …” (R068)

“The system is going haywire and it’s like ‘Hey, you know, I’m Terminator. I’m gonna do my own thing’, and then I feel unsafe forward to the point where I disengage, I turn off FSD, and I’m like ‘I don’t want to risk anything’.” (R075)

#### Unnatural automation behavior

The automation’s unnatural or non-human-like behavior contributes to human operator-initiated disengagements.

“When I feel uncomfortable, I take over quickly. It’s just … it doesn’t drive like me, which is one of the biggest problems, and one of the things that people expect from a full self-driving car, but at the moment it does not achieve that.” (R038)

“Do I disengage Autopilot, FSD Beta? FSD Beta all the time and that’s just because of how I drive. I don’t want it to do something that seems inhuman.” (R055)

“It will start doing something that at least as a human, I would not do that, and that’s typically when I would take over or override it.” (R090)

#### Random disengagements

This subcategory addresses the automation-initiated disengagements caused by system error without human operators understanding the reason for the automation-initiated disengagements.

“It just started beeping, and asking me to take control, and I could not tell what exactly it was but I just took control for 30 seconds.” (R001)

“Both of them will disengage for no reason. I’ll get the red steering wheel, and it’ll say take over immediately, and I don’t know why. I’ll be on the freeway in the middle of the night with no one around me, and the red steering wheel will come on, and it’ll tell me you take over immediately, and it’ll start slowing down for absolutely no reason. Just said take over!” (R076)

#### False positives

Human operators reported that the automation disengaged itself despite them supervising the automation.

“I rest my hand on the wheel while it’s dragging, and it will say my hand is not there. It said there is no pressure on the steering wheel so it will disable it. If somebody is not paying attention, you could cause an accident.” (R008)

“Autopilot itself will give you ‘Please keep your hands on the wheel even though your hands are on the wheel.’ It gives you that warning regardless.” (R032)

#### Unwanted action(s)

##### Operational

*Harsh deceleration*

This subcategory addresses the automation-initiated disengagements caused by harsh and sudden deceleration. Human operators associated incidences of harsh braking with the shift of the sensor suite from radar to vision and considered it a safety risk, as it could potentially lead to more incidents of the Tesla being rear-ended. After encountering incidents of harsh braking, human operators were more likely to disengage the automation.

“I was on the Interstate one time in the middle of the day, perfect weather, and it did an automatic emergency brake. People refer to that as phantom braking. It literally slammed on the brakes, and went from 80 miles an hour down to like 40 miles an hour almost instantly. So, for the next month or so I didn’t use it at all.” (R046)

“I was driving in the middle of the desert where you could see ten miles ahead. There’s no cars on a two-lane road, and it was just slamming on the brakes. So, at any time I have to be ready. It could just slam on the brakes. That’s been my recent experience with FSD Beta.” (R068)

*Erratic steering wheel movements*

Another unwanted automation behavior represents erratic steering wheel movements by FSD Beta, contributing to human operator-initiated disengagements.

“If we’re approaching a junction, it starts squirming the wheel back and forth. It does it with a lot of force. If it pulls the wheel really hard, you can stop it instantly.” (R051)

“A lot of times when I set it into FSD, and it just does dumb things like whipping in and out of lanes, I’m just gonna disengage it, and I just drive manually.” (R058)

“I only had one hand on the wheel, my elbow was on the armrest, and it swerved. It made this maneuver so strong that it actually bent my wrist down, pulled my arm off the armrest, and I was shocked. I saved the video from the dashcam thing. ‘Maybe I missed something. Maybe there was a person that I didn’t see.’ There was nothing. There was absolutely no reason for it to do that maneuver.” (R096)

*Steering into adjacent traffic*

The occurrence of FSD Beta steering towards neighboring traffic represents an unintended automated system behavior contributing to human operator-initiated disengagements of the automation.

“It was just a straight road. All it needed to do was just drive straight. Stay in the lane lines. Don’t hit the car in front of me. Maintain speed. Instead, it tried to swerve left into a vehicle next to me, so I had to disengage.” (R007)

“The very first time I used FSD Beta, I turned it on my street. It goes down, and it tries to veer into a car immediately, and I was like ‘Ohh no, this isn’t good.’” (R066)

“FSD Beta is downright scary. It almost makes you piss your pants when it jerks out of a lane or jerks into a lane. You get into an accident, hurt yourself or kill somebody or kill yourself or whatever. There’s nothing positive about that.” (R069)

##### Tactical

*Undesired lane changes*

This subcategory addresses human operator-initiated disengagements caused by the automation performing unexpected, unnecessary, too aggressive, or too conservative lane changes.

“FSD Beta ‒ it just changed lanes on its own as a motorcycle was rapidly approaching. I don’t want to kill the motorcyclists. It’s like ‘Oh, I need to change lanes.’ It randomly decided to change lanes. Didn’t give me time to do a head check because it just goes as soon as it wants to. Why are we changing lanes? It was unnecessary. It was unexpected. I did the head check, saw the motorcycle in my rearview mirror rapidly approaching, and took over.” (R007)

“I was up in North Carolina on FSD Beta, and it said ‘I wanna get in the left lane’ so I thought ‘Well, that’s weird. There’s nobody around. We don’t need to be in the left lane’, so I corrected it and made it go back to the right lane, and it did it again, and I corrected it again, and then this poor lady drives up in the lane beside me, and is trying to pass me in it, and it tries to do it again, and I said ‘No, this is not O.K.’, and I turned it off. So.” (R100)

*Misidentifying correct lane*

This subcategory addresses human operator-initiated disengagements caused by FSD Beta driving into bike, bus, or parking lanes.

“They just need to program it so it can read the word ‘bus lane’. It wants to go into that right lane, every time into the bus lane, and I have to cancel it.” (R081)

“I will disengage if it’s not handling an area correctly. The white bike lanes, the car will sometimes attempt to go there, and if I see it happening, I will disengage FSD, report it, because it shouldn’t be doing that.” (R094)

*Creeping*

One of the main reasons for human operator-initiated disengagements at intersections was due to the creeping behavior of FSD Beta contributing to confusion and annoyance among other road users.

“The big reason that I don’t use it all the time is just there’s all these social norms regarding how people drive, and people do not like it when you drive differently than they do. The car is pausing at a four way stop and then slowly, inexplicably creeping through the intersection when it’s clearly time to go. People stop, and they look at you as you drive past, raise both hands. ‘Go!’ ‘Hey, look, no hands!” (R068)

“When you’re coming up to a stop sign, it does this thing called creeping. It does stop, and then it creeps forward slowly to make sure it’s safe to go through the intersection. There is nobody in any direction, and it just sits there, and I just nudged the accelerator pedal, just tap it a little bit, and then it’s like ‘Ohh, ok’, and then it’ll finally go, and make the left turn.” (R069)

*Rolling through stop signs*

Human operators mentioned the National Highway Traffic Safety Administration’s (NHTSA) efforts to regulate FSD Beta’s practice of ‘rolling through stop signs’, which, although it represents an unlawful action, is a frequently observed human behavior in certain regions.

“We’re already dealing with the National Highway Traffic Safety Administration regulating Tesla. Beta would do rolling stops at stop signs and depending on where you live, that’s a natural normal thing to do, but now Autopilot always has to stop at stop signs, and that makes the driving experience less natural.” (R038)

“NHTSA disabled the rolling stock functionalities of FSD Beta. It makes the car fully stop, which causes a huge traffic delay. If there’s a clear intersection, almost everyone does rolling stops. We have no rolling stops, which makes the car sit at a stop sign for a significantly amount of time. So, I’m finding myself using the Beta less.” (R094)

*Running red lights/stop signs*

This subcategory addresses human operator- and automation-initiated disengagements resulting from FSD Beta running a red light or a stop sign.

“It actually has tried running a red light for me. I was following one of the test cars for one of the self-driving car companies, which ran a very late yellow, but then my car followed and when the light turned red, it wasn’t slowing down. It wasn’t reacting to it at all, so I took over.” (R003)

“You would come up to the stop sign, you would slow down to stop, and it just kept going. It was just gonna go right through it, and that’s when I hit the brake. So, if I had not hit the brake, it would have gone right through that stop.” (R069)

*Unprotected left-turns*

Human operators reported to disengage FSD Beta during unprotected left-hand turns. Such turns involve turning left at an intersection with a green light, instead of a green arrow, while oncoming traffic has the right of way.

“Waiting to turn unprotected left, and I’m waiting for it to decide what it’s going to do. I have my foot right over the brake pedal, and the car just suddenly tries to go. It just feels like it’s jumping. These cars that are coming straight toward me, and a person wouldn’t do that, … unless they’re trying to die.” (R018)

“The unprotected left turns don’t work. It’s kind of scary. The car will kind of creep forward, and it will wait, and when the car passes by, it’s still kind of thinking. ‘Should I go? Should I stay?’, and then it slowly starts moving.” (R058)

*Other turning situations*

Human operators also indicated to disengage the automation in other turning situations, such as ‘turning-on-red’, signalized left- and right-hand turning, and U-turning situations. In ‘turning-on red’ situations, cars are allowed to turn into the direction of traffic at a traffic light showing a right signal^37^. Human operators reported that FSD Beta did not detect these traffic signs, resulting in human operators disengaging the automation to avoid annoying other drivers or losing time while traveling.

“FSD Beta doesn’t recognize those ‘turn on red’ signs, so previously with FSD stopping at red lights, you had to manually intervene to make it proceed on green, and now I often have to manually intervene on red.” (R007)

“I stopped at a stop sign and there’s a highway in front. There are trees in the way. It decided that there were no cars coming and started to move out full speed into the highway, and there was another car coming. I had to slam on the brakes because the other car might have run into me.” (R021)

“Full Self Driving Beta ‒ it doesn’t know how to make turns safely. Sometimes, it takes some weirdly fast. It’ll stop. ‘OK, when it’s time to go?’, and I’ll be ‘OK, now!’, and then it’ll go really fast. It’s like ‘You could have just slowed down, and done that turn slowly.’ So, it’s not super safe.” (R059)

##### Strategic

Route unfamiliarity

This subcategory addresses human operator-initiated disengagements on unfamiliar roads due to their lack of trust in the automation’s capability to handle these areas safely given their lack of experience with the automation in these areas, and a desire to be in control.

“If I’m in a situation where I don’t drive very often, then I’m likely to keep FSD Beta on a very short leash. Once it gets in a crowded situation coming through, or it starts acting a little wonky, I’m gonna take it down, and say ‘Hey, I’m not comfortable using it there. I haven’t tested it in this environment enough to feel comfortable doing it.’” (R026)

“So, for Autopilot, I engaged it during interchanges, or maybe an exit, or there’s an unfamiliar area, and I wanna stay in control. If I’m not comfortable in an area, I will definitely not let Autopilot take the lead.” (R094)

*Taking the wrong route*

This subcategory addresses human operator-initiated disengagements caused by the automation taking the wrong route. These errors are attributed to a misalignment of mapping and navigation data.

“It sometimes will do really dumb directions. It’s like ‘Just turn left here. You don’t need to go right, and go around the square and then come back around.’ You can’t easily necessarily correct that in the moment, so those are instances where you have to disengage.” (R007)

“The majority of my disengagements in 10.2 are related to navigation, and so I disengage to go to the actual destination that I’m going to.” (R011)

“One of the problem areas is that sometimes the map data and information associated with FSD Beta is not the most accurate, and you may have to take over in order to get to the correct location.” (R037)

### Human operator’s perception of other humans

Human operators also reported to disengage the automation when they perceived that other humans, such as passengers and road users, were uncomfortable with and frustrated by the automation’s behavior.

#### Passenger discomfort

Human operators initiated the disengagement in response to passengers’ discomfort and lack of trust in the car with Autopilot and FSD Beta engaged. Reasons for this included a perceived lack of control and the automation’s erratic, harsh, and unpredictable behavior.

“I am less likely to use it when I have passengers in the car, when I have a car full of family, and passengers who are like ‘Oh, what’s that thing been about? Why is it beeping at you?’, or if I need to make a sudden correction, I don’t wanna do that when I have got passengers that might be discomforted from that.” (R027)

“It’s my experience in my view and my feelings, and then that of my wife. I’ve had three occasions where had I not been absolutely paying attention and being fully in control of the car, it would have caused an accident. My wife’s comment is ‘If I use the FSD Beta with her in the car again, then I should just go get the divorce papers.’ It’s that bad.” (R069)

#### (Anticipated) frustration

This subcategory addresses human operator-initiated disengagements to avoid confusion and rage of other road users interacting with Autopilot and FSD Beta on public roads.

“The second biggest risk is road rage. That computer does weird things and it just makes them really angry, and then they are angry at you. That’s actually been my biggest issue with the system. I’ll just turn the system off. Anytime someone following me closely, I’m nervous, this system is going to produce either an accident or anger.” (R002)

“Most of my disengagements are because I don’t like how I perceive it will make other drivers feel because I’m a very courteous driver. When Autopilot starts creeping out, I do feel it’s going to move towards them, and that’s going to make them feel I might dart out, and so how are they gonna react?” (R085)

#### Reckless behavior

Another reason for disengaging the automation was the reckless behavior of other drivers, such as drivers following too closely from behind or swerving into the lane of the Tesla.

“It was only at one time that I was on the Autopilot, and a car merged in so quickly that I actually had to come take control of the vehicle because my vehicle was not going to stop because he just merged out of nowhere.” (R020)

“We had about four incidents where we had 18 wheelers cross over the line. So, it’s something you want to keep an eye on to make sure that they’re not going to come over in the lane.” (R024)

### Automation’s perception of human operator

The automation initiated the disengagement itself if it perceived human operators becoming complacent. Both the automation and human operators initiated the disengagement when human operators provided an inappropriate amount of torque to the steering wheel, or when they exceeded the speed limit.

#### Complacency

Complacency contributed to the automation initiating the disengagement.

“When I first used the car, I was just not paying attention, and it threw me off because I wasn’t paying attention.‬” (R066)

“There’s been a few times I forgot to do the nudging. I was listening to music or on a phone call or something, and then it will get mad at you, and beep, and then it’ll kick you out. It just tells you to take over.” (R070)

#### Inappropriate amount of torque applied to steering wheel

Human operators applying an inappropriate amount of torque to the steering wheel resulted in unintentionally disengaging the automation.

“Full Self-driving Beta is really easy for taking over because you keep your hands on the steering wheel, and if it jerks the steering wheel with your hands on it, you’re just gonna automatically disengage.” (R049)

“It’s very hard to sometimes not accidentally disengage Autopilot when you’re trying to show the car that I have my hands on the steering wheel.” (R088)

#### Speed rule violation

This subcategory addresses human operator- and automation-initiated disengagements that occurred when human operators exceeded the speed limit. Human operators reported to disengage the automation to exceed the speed limit for reasons of safety and efficiency, e.g., to overtake a slower vehicle, or a lack of knowledge.

“Where I need to go above the speed limit to get around a car safely, you have to disengage Autopilot because it will not do that for you. Autopilot currently does not have the ability of going above its speed to get out of a bad situation.” (R031)

“It used to kick off if you hit 80 miles an hour, and in Arizona, if you’re doing 80 miles an hour, you’re basically in people’s way. So one time I was trying to get by a truck, and you get these flashing lights, and it kicks you off, and you’re in jail until you stop, restart the car. You can’t just kick it back in.” (R082)

### Automation incapability in environment

Both the automation and human operators initiated the disengagement in environments exceeding the perceived capabilities of the automation.

#### Weather

This category addresses human operator- and automation-initiated disengagements in inclement weather conditions, with human operators disengaging the automation, and the automation asking human operators to take back control in poor visibility conditions given the current limitations of the sensor suite.

“Autopilot heavily relies on all the cameras, and we have very bad weather here in Canada. When the cameras gonna cover it up too much with the snow, it will sometimes abruptly disable itself. I’ve never had it really lose control because it’s slippery roads, but it’s always in the back of my mind in bad weather.” (R016)

“In really bad weather, Autopilot is going to start chirping and making noises to tell you to take over when water gets on all the cameras, and it can’t see any longer. I’ll just turn it off until I’m done driving through the rainstorm.” (R070)

#### Non-standard roads

This category addresses human operator- and automation-initiated disengagements due to inconsistent lane markings and differences in lane width, which often resulted in the car re-centering itself within the lane.

“If you encounter an area where the paint disappears, the car tries to center itself in. Now if it’s a two-lane highway, it may try to center itself, which is nerve wracking. I’ve actually turned it off to get past those areas.” (R056)

“Earlier when I came to an on-ramp, off-ramp where there was no line, it could not see the line properly and then it disengaged, and the alarm went on, and it started slowing down. So, I took over.” (R073)

#### Curves, hills

This subcategory addresses human operator- and automation-initiated disengagements in curves (sharp turns) and on hills.

“The largest issues I’ve historically had with Autopilot is, it doesn’t turn sharply enough on sharp turns, so I get quite a bit of anxiety justifiably going into sharper turns. I usually would disengage on sharper turns just because it wouldn’t be steering enough.” (R007)

“There are certain sections of freeway where there are tight turns when everyone is going really fast, and I’ll just manually take over on the really hard turns.” (R042)

#### Object detection

This subcategory addresses human operator- and automation-initiated disengagements in response to stationary and non-stationary objects and events in the environment. Human operators decided to disengage the automation in these situations because the automation failed to detect and maneuver around these objects, or because of the automation’s lane centering, which resulted in an uncomfortable close proximity to these objects.

#### Stationary objects

Stationary objects and events in the environment included potholes, parked cars, road debris, construction, cones, bushes / trees, buildings, gates, and railway crossings.

“So, there’s an object in the road I didn’t see until my fiancé said ‘Hey, there’s something in the road’. You can see that there’s nothing visual here. So, I would have hit that if I didn’t take over.” (R011)

“Full Self-Driving Beta drove me home, and everything was fine until it almost drove into a building. I had to grab the wheel, put on the brakes.” (R018)

“There was a train going through. I approached the stop manually, and decided to turn Beta, and what it was displaying on the screen was a line of semi-trucks, and then the visualization disappeared, and it started to accelerate, and there’s a train creeping forward like full normal acceleration. I’m not gonna let it get very close to a moving train. So, I hit the brakes.” (R096)

#### Non-stationary objects

Human operators reported to disengage the automation in situations with non-stationary objects and events in the environment, such as other vehicles in adjacent lanes, emergency vehicles, and vulnerable road users.

“A woman in the crosswalk was waiting for the walk indicator, and I’m waiting to turn right. I’m on Full Self Driving, and I’m looking at her. She’s just waiting for the light. I think ‘My car’s gonna try to hit her, I know. I was gonna try to hit her, I know.’ The light turns green. Ohh. Turned to hit her.” (R018)

“It is not that great at picking up on emergency vehicles yet, so if I see a police officer off the distance with its flashing lights, it will start to slow down usually way too aggressively. Other people around me are not slowing down yet. I usually have to take over in those situations.” (R074)

“FSD Beta ‒ whenever there are many turns with lots of pedestrians, I’m not going to. It’s too scary. I don’t want it to hit anyone. I have to disable it.” (R074)

#### Intersections

This subcategory addresses human operator- and automation-initiated disengagements in intersections, including roundabouts. Human operators reported to disengage the automation due to its limitations in handling intersections safely or their fear of getting rear-ended due to unexpected or unnatural system behavior. Human operators also reported to disengage FSD Beta due to the system’s lack of negotiation and interaction skills with other road users.

“Roundabouts are a very challenging scenario. FSD Beta will almost always come to a complete stop at a roundabout or before, and so I feel a sense of social anxiety if there’s a car behind me, and the car just completely comes to a stop. I will tap on the accelerator to encourage the car to go through the roundabout.” (R050)

“If I’m at an intersection, it can be too slow, and there’s a lot of cars behind me, and I’m like ‘Come on, no one’s there. Come on. Accelerate because you can do that. You can accelerate.’ It’ll go and it’ll do the turn. I don’t always do that because certain intersections are too big. So, then I’ll just disengage, do the difficult spot, and re-engage.” (R052)

“Every intersection I’m like, ‘OK, we’re gonna do this. Right. Great. Wonderful. Doing good. Next one. You can do this. Right. OK, good. Ohh. There’s a little conservative here. Let me flag this.’ Then the next time, I know, ‘OK, it might be a little conservative here. It might get confused, and be prepared to take over.’” (R057)

#### Discontinuities in road design

This subcategory addresses human operator- and automation-initiated disengagements due to discontinuities in road design, such as in on- and off-ramp, lane merging, and splitting situations.

“You’ll be driving along, and a new lane opens up, and the car just went into the left turn lane for no reason at all so I immediately intervened, and took it to the correct lane.” (R010)

“When I took the exit on the freeway, when it just shouldn’t, I didn’t use it for the rest of the 30-minute drive. I just was like ‘No, I am not using this because we almost just got in an accident.’” (R043)

“It makes the maneuver way too aggressively, basically whipping you into that lane, and I’m not joking when I use the word ‘whipping you’ because you’re there like ‘Hey, lane change’. It literally throws you like this as it makes the maneuvering to the other lane. I have to disable it every time it needs to take that style of exit.” (R071)

#### Complex, heavy traffic

This subcategory addresses human operator- initiated disengagements in complex and heavy-traffic situations.

“If there’s a lot of cars on the road, I do not use Beta, and why is obviously because sometimes it tries to kill me.” (R023)

“If there’s a lot of traffic, I probably won’t use it because I don’t want to intervene with the traffic, but if it’s empty, it’s a dead area, then I’m gonna use it.” (R048)

### Synthesis

**Figure 1:**
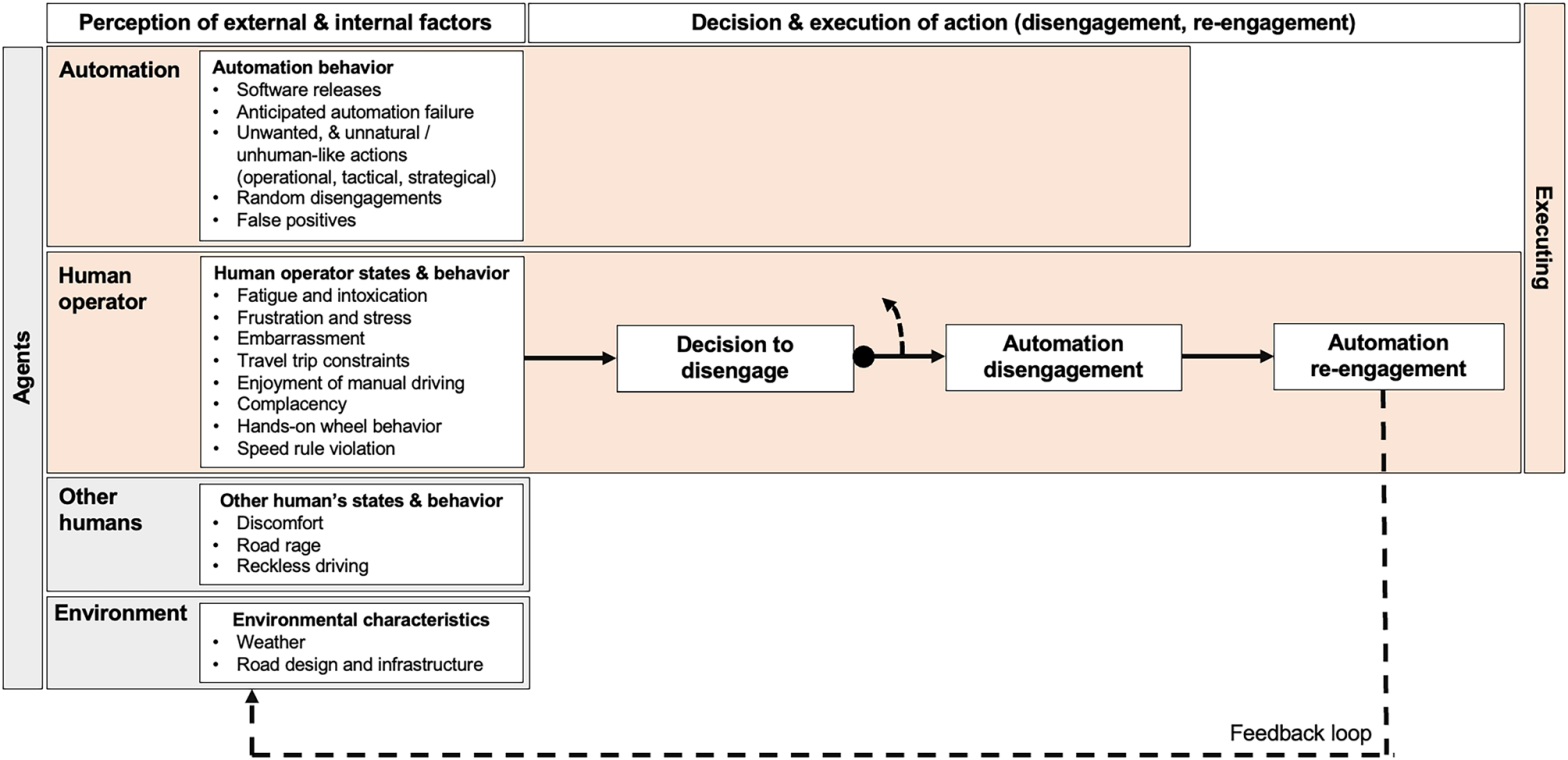
Conceptual framework for automation disengagements

The results of the data analysis inform the development of a conceptual framework for automation disengagements, as presented in Fig. 1. The framework posits that both the automation and human operators (i.e., drivers) can initiate the disengagement. Both agents assess each other’s performance and capabilities in the given environment. The framework identifies that adverse human operator states and travel trip as well as time constraints contributed to human operator-initiated disengagements. Certain (anticipated) automation behaviors resulted in negative states experienced by the human operator, such as frustration, stress, and embarrassment. Human operators decided to disengage the automation in anticipation of automation failure in environments exceeding the automation’s capabilities or when the automation exhibits unnatural or unwanted behavior. Human operators also initiated the disengagement when other road users were uncomfortable or frustrated by the automation’s actions. Similarly, the automation perceived human operators as insufficiently vigilant (based on the vehicle’s driver monitoring systems), violating speed limit rules, or it assessed its own capabilities as inadequate in the given environment, leading to self-disengagement. The framework also includes a feedback loop, which pertains to the re-engagement of the automation after its disengagement by human operators, which influences the perception of the external and internal factors by human operators and the automation.

## Discussion

This study presents the findings of semi-structured interviews conducted with 103 users of Tesla’s Autopilot and FSD Beta system. Our analysis of the interview data led to the extraction of a variety of reasons for automation disengagement, both automation-initiated and human operator-initiated. In turn, an analysis of these different reasons for disengagement has informed the development of a conceptual framework, which posits that human operators and the automation both perceive the performance and capability of the other agent. The reasons contributing to human operator-initiated disengagements pertained to the human operator itself, the human operator’s perception of the automation and other road users, as well as the automation’s perception of the human operator.

### Conceptual framework for automation disengagements

The categories informed the development of a conceptual framework for automation disengagements, which posits that human operators and the automation can perceive the performance and capability of the other agent, initiating the disengagement accordingly. The idea that both human operators and the automation can initiate the disengagement is consistent with existing literature^16,38^. Human operators may initiate the disengagement if they perceive the automation’s capabilities to be lower than their own in handling a maneuver safely.

Our results and framework consider not only the impact of automation behavior and environmental characteristics, such as road infrastructure and design, on human operators but also the impact of other humans (e.g., passengers, other road users). Previous driver behavior and automation models primarily featured the driver and automation as main actors^39–41^. Our framework resonates with the principle of distributed cognition, which states that information does not only exist in the minds of individuals but also in the objects and artefacts that individuals use as well as in the interactions between individuals and objects and artefacts^27^. As road traffic increasingly incorporates sensors, computers, and communication systems, the concept of distributed cognition is expected to become even more important in understanding and managing interactions within the transportation system.

### Human operator’s states

Our research demonstrates that human operators opt to disengage the automation due to the human operator’s state of mind. One reason for disengagement is that some human operators like the occasional joy of manual driving^42^. Negative human operator states contributing to human operator-initiated disengagements that were observed are frustration, stress, and embarrassment. While some constructs such as trust, frustration, mental workload, and situational awareness have already been treated in the literature^2,3,16,21,42,43^, our research is unique in that it highlights that embarrassment about the vehicle’s behavior is an important psychological state correlating with automation disengagements.

### Human operator’s perception of automation

Human operators and the automation may initiate the disengagement in anticipation of automation failure in situations exceeding the perceived capability of the automation. Studies have shown that drivers frequently disengage the automation in an anticipatory or precautionary manner^16,22,24,38,44^. Some human operator-initiated disengagements might have been unnecessary, as it remains unclear whether the system would have handled the situation safely without intervention by human operators. Consistent with^22^, human operators indicated initiating the disengagement not to mitigate immediate risk but to perform tactical maneuvers exceeding the automation’s capabilities (e.g., passing a vehicle in front).

The interviews have also shown that human operators may opt to disengage the automation when it demonstrates unnatural, non-human-like, or undesirable behavior at the operational, tactical, or strategic levels of decision-making^40^, such as harsh deceleration or phantom braking, erratic steering wheel movements, steering into adjacent traffic, choosing the incorrect route, unanticipated lane changes, misidentifying the correct lane, or creeping at intersections. While the phantom braking behavior associated with assisted and partially automated driving represents a known vehicle behavior^45^, our study offers new insights into additional vehicle behaviors (e.g., steering into oncoming traffic, undesired lane changes). Some of these behaviors are safety–critical and could have produced an accident if human operators had not intervened. Our research has also revealed unexpected automation-initiated disengagements, also known as “silent failures”^46^, without drivers understanding the reasons underlying these disengagements. Research has shown that unexpected disengagements could have severe safety implications if they remain unnoticed by complacent, distracted drivers, failing to intervene in time^47^. Potentially safety–critical system behaviors (e.g., steering into oncoming traffic) could be mitigated through improvements in automated driving design. These observations relate to the operational design domain (ODD) concept, which describes the conditions under which automated vehicles can function^48^. Our study has shown that weather conditions and road infrastructure and design characteristics have contributed to disengagements initiated by both the automation and human operators. The incapability of the automation in these environments supports existing research on environmental factors contributing to automation disengagement, as well as the need for human operator intervention in challenging situations^14,16,44,49^. Some of these automation disengagements could be prevented by improvements in the sensor suite, and road infrastructure and design. For example, road maintenance could reduce the occurrence of automation disengagements^16^.

### Human operator’s perception of other humans

Our study emphasizes the significant role other humans play in shaping disengagements of automation. Human operators may choose to disengage the automation if passengers and other road users feel uncomfortable or are angry due to negative automation behavior. Future research should examine the notion of the ‘theory of mind ability’^50,51^ in the context of automation disengagements to infer how the perception of other humans by human operators influence the disengagement of the automation by human operators. The perceived risk of accidents and loss of control may be higher for passengers than for drivers^52^, and other road users who lack direct control over the actions of the automation. Our research suggests that if other humans are uncomfortable with human operators engaging the automation, human operators might opt to use the system alone without passengers onboard, testing and experiencing its limits, leading to an increase in the single-person vehicle miles traveled (see^1^). Car manufacturers should consider the impact of automation on passengers and other road users, designing the human–machine interaction in such a way that perceived safety and trust of humans inside and outside automated vehicles is promoted, and frustration and stress reduced.

### Automation’s perception of human operator

Our study has also revealed that the automation decides to initiate the disengagement if it, based on the vehicle’s driver monitoring systems, detects that the human operator becomes complacent or violates traffic rules. Driver inattention or distraction has contributed to automation-initiated disengagements (or system lockouts) in the study of^53^ conducted with drivers using partial driving automation. Torque-based steering wheel monitoring systems are not fully effective in detecting complacency, as they can be manipulated by operators placing objects on the wheel to mimic engagement^1^. In addition to robust driver monitoring systems, a holistic approach to driver management, the development of proactive strategies for driver engagement, and support in understanding and using the systems safely may be needed^24^. The risk of complacency associated with partial driving automation is well-known^3,6,21,47^. Analogous to the concepts of ‘trust in automation’ and ‘trust in self’, the automation should have accurate trust in humans and trust in self, initiating the disengagement when the perceived reliability of itself is lower than the perceived reliability of human control. Education and training could play a crucial role in raising awareness of system limitations and improving system handling (e.g., the required amount of torque applied to the steering wheel). However, education and training may be insufficient to prevent the occurrence of misuse of automation. Complacency might be automation-induced to some extent. The notion of automation-induced complacency was studied in the context of automated aircraft, with the pilot and crew failing to monitor the automation in highly reliable automated environments^54^. Boelhouwer et al.^55^ have shown that the provision of system information did not support drivers in their decision to take over control from the partially automated car, or to rely on the system, and respondents’ mental model was not sufficiently changed by the information.

### Limitations and implications for future research

There are several limitations of the present study, providing important avenues for future research.

First, the data on human operator- and automation-initiated disengagements reflects the subjective perceptions of respondents. This implies that the present study has not examined whether respondents correctly identified the reasons for the disengagement.

Second, we have not objectively verified whether respondents were engaged in secondary activities prior to the disengagement.

Third, while disengagements are considered a safety risk^16^, it is not clear to what extent disengaging the automation contributes to a decrease or increase in accident risk compared to not disengaging the automation.

Fourth, in line with^22^, our sample consists of a relatively small number of early adopters self-selecting themselves into the study who may not be representative of the broader population of users of partially automated cars. It is unclear to what extent our findings can be generalized to respondents differing in gender, age, social norms, or cultural values^7^. We recommend future research to perform studies in naturalistic driving conditions to investigate to what extent disengagements influence accident risk with a representative population of human operators of partially automated cars.

Fifth, we have not examined how longer-term use of the automation contributes to initiating the disengagement. Longer-term studies should be conducted to examine changes in learning and behaviors contributing to disengagements over time, investigating the rare types of disengagements^22^.

Sixth, we have limited our analysis to Tesla users. Future research should investigate to what extent the characteristics of different partially automated driving systems influence automation disengagements^22^.

## Conclusion

The study presents the results of semi-structured interviews with 103 users of Tesla’s full self-driving (FSD) Beta system and standard Autopilot, exploring the factors contributing to human operator- and automation-initiated disengagements of both systems. The study proposes a novel conceptual framework for automation- and human operator-initiated disengagements, which takes into account the impact of automation behavior not only on the human operators of automation but also on passengers and other road users. The results have shown that the contributing factors leading to human operator- and automation-initiated disengagement of Autopilot and FSD Beta pertain to human operator states, safety–critical system behaviors (e.g., steering into oncoming traffic), other road users’ behavior (e.g., road rage, reckless driving behavior), and road infrastructure and design factors (e.g., missing lane markings). The findings provide new insights into the factors contributing to disengaging partial driving automation, with valuable information on potential edge cases of automated vehicle technology. Our paper emphasizes that automation disengagement is not solely a human operator-based concern but also a social phenomenon, in which the operator may feel a sense of embarrassment or self-consciousness about the impact of the automation on other road users. This social dimension behind automation disengagements highlights the importance of considering the experiences and perceptions of other road users in addition to the human operator when evaluating the effectiveness and usability of automation systems.

### Supplementary Information

Supplementary Information 1.

Supplementary Information 2.

## Data Availability

The datasets generated and/or analysed during the current study are not publicly available as a portion of the dataset is currently being analysed in another study. The data will be published after the corresponding publications have been submitted, and are available to the public. To obtain access to the data, the corresponding author can be contacted at s.nordhoff@tudelft.nl.
