# Supplementary material for: A conceptual framework for automation disengagements
Source: Sci Rep. 2024 Apr 15;14:8654. doi: 10.1038/s41598-024-57882-6 (PMC11018869; doi:10.1038/s41598-024-57882-6)
Supplement: Supplementary file 1 — Supplementary Information 1. [file 41598_2024_57882_MOESM1_ESM.docx]

1. **Appendix**

*Table 1A.* Overview of questionnaire

| **Question number** | **Question** |
| --- | --- |
| Q1 | Do you have the Full Self-Driving Beta (FSD Beta) feature? (1 = Yes, 2 = No) |
| Q2 | Before the first time of using Autopilot and FSD Beta, did you watch / read / listen to information on how to use it? (1 = Yes, 2 = No) |
| Q3 | Please mention the type of information you consulted on how to use Autopilot and FSD Beta (website of Tesla ([www.tesla.com](http://www.tesla.com)), car dealer / sales point, online communities and forums, YouTube videos, newspapers and magazines, friends, family, colleagues, driver manual) |
| Q4 | Please describe your experience with using Autopilot and FSD Beta and the benefits and risks associated with using it. Please explain your answer. |
| Q5 | Have your expectations of using Autopilot and FSD Beta been fulfilled? Why / why not? |
| Q6 | Why do you use Autopilot and FSD Beta? |
| Q7 | Did you ever stop using Autopilot and FSD Beta (for prolonged periods of time)? |
| **Next, we would like to explore your perceptions regarding four general statements about the operation of Autopilot and FSD Beta.** | |
| Q8 | The current Autopilot does make driving autonomous. Is that correct? (1 = Yes, 2 = No, 3 = I don’t know) |
| Q9 | There are no safety issues with Autopilot. Is that correct? (1 = Yes, 2 = No, 3 = I don’t know) |
| Q10 | Autopilot is a hands-on feature. Is that correct? (1 = Yes, 2 = No, 3 = I don’t know) |
| Q11 | Tesla FSD Beta is safer than a human. Is that correct? (1 = Yes, 2 = No, 3 = I don’t know) |
| **With the next section, we would like to explore your perceptions of safety while using Autopilot and FSD Beta.** | |
| Q12 | Do you feel safe when Autopilot and FSD Beta is active? Why / why not? |
| Q13 | What / how do you feel when you feel safe / unsafe? Please explain. |
| Q14 | What is it about Autopilot and FSD Beta that is safe / unsafe? Please explain. |
| Q15 | Now please remember the situation / s in which you typically feel unsafe when Autopilot and FSD Beta is active and describe these situations. |
| Q16 | What can Autopilot and FSD Beta do to support your safety in Autopilot and FSD Beta? Please explain. |
| Q17 | Does feeling safe / feeling unsafe impact how you use Autopilot and FSD Beta on your next drives / in the future? Please explain. |
| Q18 | Has your perceived safety changed over time? If so, how? |
| **With the next section, we would like to explore your trust in Autopilot and FSD Beta.** | |
| Q19 | How would you position your level of trust in Autopilot and FSD Beta. (1 = I don’t trust it at all, 2 = I don’t trust it, 3 = I neither don’t trust it at all nor trust it a lot, 4 = I trust it, 5 = I trust it a lot) |
| Q20 | What can Autopilot and FSD Beta do to support your trust in Autopilot and FSD Beta? |
| Q21 | Does your trust / distrust in Autopilot and FSD Beta impact how you use Autopilot and FSD Beta on your next drives / in the future? Please explain. |
| Q22 | Has your trust changed over time? If so, how? |
| Q23 | When you do compare yourself with other drivers, Autopilot, and FSD Beta, do you think you are ...  (1 = A much worse driver, 2 = A worse driver, 3 = Not a better nor a worse driver, 4 = A better driver, 5 = A much better driver) (De Craen, 2010) |
| **With the next section, we would like to explore how you typically use Autopilot and FSD Beta.** | |
| Q24 | How do you typically place your hands on the steering wheel when Autopilot and FSD Beta is active? Please select the image that serves as best representation of your placement of your hands on the steering wheel when Autopilot / FSD Beta is active and explain your answer.  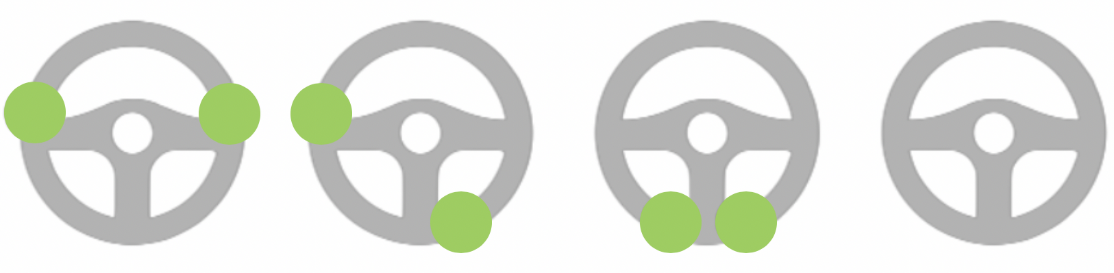  Figure is from Morando et al. (2021) |
| Q25 | Do you typically keep your hands on the steering wheel at all times? |
| Q26 | Are you typically fully attentive and alert at all times? |
| Q27 | How often do you typically engage in other secondary activities while Autopilot and FSD Beta is active? (Never, rarely, occasionally, frequently, always; monitoring the road ahead, talking to fellow travelers, observing the landscape, using the phone for music selection, using the phone for navigation, using the phone for calls, eating and drinking, using the phone for texting, watching videos / TV shows, sleeping) |
| Q28 | Do you disengage Autopilot and FSD Beta? Why / why not? |
| Q29 | Does Autopilot and FSD Beta disengage? When / in which situations? |
| Q30 | How do you typically place your eyes when Autopilot and FSD Beta is active? |
| Q31 | Do you typically keep your eyes on the road at all times? |
| Q32 | Do you typically monitor the vehicle and its surroundings at all times? |
| Q33 | How do you typically place your feet when Autopilot and FSD Beta is active? |
| Q34 | Do you typically stay prepared to take corrective actions at all times? |
| Q35 | Has your use of Autopilot (in terms of how you placed your hands on the steering wheel, eyes on the road, and feet) changed over time? If so, how? |

*Table 1B.* Overview of seed terms and exemplary quotes representing sub-categories

| **Sub-category** | **Seed terms** | **Quotes** |
| --- | --- | --- |
| **Disengagement** | Disengage, disengaged, disable, disabled, correct, corrected, intervene, intervened, disuse, disused, switch off, switched off, deactivate, deactivated, stop using, stopped using, didn't use, can’t use, don’t use, not use, turn it off, turned it off, turn off, turned off, take over, took over, cancel, cancelled, won’t use | - |
| **Human operator state** | |  |
| Fatigue and intoxication | Tired, fatigued, medication, medicated, fatigue, alcohol, impaired, impairment, intoxicated, intoxication | “I stopped using FSD Beta when I’m fatigued, and I just don’t feel like paying that close attention.” (R012)  “If I’m tired at the end of the day, I’ll just turn off the Beta and drive myself because I need to pay attention.’” (R017) |
| Frustration and stress | Frustration, frustrated, stressed, stress | “There is something in that drive that just gets me to say ‘I’m done’. I’m gonna make sure that I don’t get frustrated, and that it doesn’t make a mistake.” (R061)  “With the Beta, I was frustrated. I just get angry, and I’m like ‘You know what? This is not even usable.’ So, I just stopped using it for a while.” (R096) |
| Embarassment | Shame, ashamed, embarrassment, embarrassed, embarrassing, awkward | “If I allowed to do auto lane change, sometimes I will interject because it’ll be like ‘I wanna change back lanes’ but then I know 5 seconds later, it’s gonna come back out of that driving lane again to pass another vehicle. So sometimes I may turn off the auto lane change feature versus me look like a crazy driver just zipping back and forth.” (R071)  “When it continues to go, it already stopped but the person is thanking me for stopping, and in that time that the person thanks me, the car decides ‘Well, if you’re not gonna go, I’m just going to go instead.’ It confuses the person. It makes me look bad. It’s just bad.” (R074) |
| Travel trip constraints | Hurry, parking, pull in, pull out, driveway | “If I’m in a little bit of a hurry, I’ll take over myself because it is probably slower. It’s more careful and cautious than I would in some ways so if I’m trying to save time, I’ll take over myself.” (R021)  “I will disable it if I need to get to my destination on a less relaxed timeline, because FSD is great for obeying all the laws that it’s supposed to, and there are times where you’re running late, you’re gonna be in a little bit of a hurry.” (R061) |
| Enjoy manual driving | Enjoy driving, fun, enjoyment, joy | “I will disengage Autopilot is if I want to have a little bit of fun. The car will never use its full acceleration capability when it’s in Autopilot or Full Self-Driving, and so if I’m the lead person at a stoplight, and there’s nobody that’s going to run a red, I’ll turn off Autopilot so that I can be more aggressive, and then turn it back on once we’re into our normal speeds.” (R037)  “Almost the only time I ever turn it off is if I’m doing a joyride, or in the mountains, and really wanna experience the driving experience. Similar to some of the same motivations I use whenever I do auto cross or rally cross type of stuff as a hobby on closed tracks.” (R065) |
| Software releases | Software release, software releases, update, updates, release, releases, bug, bugs | “If there is a bad update, I probably just wouldn’t use it at all. If they released an update that just completely broke something, I probably just would stop using it until the next update.” (R063)  “I don’t use it every single day because without another update, it pretty much acts the same way. So, unless I don’t have an update, there’s no sense for me to keep driving the same area. I’m not gonna get a different result.” (R094) |
| Anticipated automation failure | Before, accident, accidents, collision, collisions, uncomfortable, discomfort, unsafe, feel unsafe, feeling safe, don't trust, didn’t trust, trust | “Sometimes there’s a hole in traffic, and you can make a left turn. So, the question is how? Is the car going to accelerate through that hole in a way I’m comfortable with, and sometimes I’m just not willing to find out. I’m gonna put my foot on the accelerator because finding out the answer to this question is very significant. It’s an accident.” (R012)  “The first times I showed it to friends and family, it tried to kill us a couple times, and that’s part of the experience. If I just completely let go that wheel, it probably would have done something that would have caused an accident.” (R074) |
| Unnatural / unhuman automation behavior | Natural, unnatural, human, not human, unhuman | “I’m not always sure that’s going to do the right thing. It doesn’t always do it soon or not soon, or at least as early as I would do it myself so there’s that divergent between what I would do and what the car is doing. I pretty much always take over at that point.” (R003)  “When there’s no risk in these scenarios, the car sometimes will do behaviors that are like ‘OK, so why did you just sort of come to a stop right here?’ If there were other cars around, I’m gonna wanna intervene.” (R012) |
| Random disengagements | Why, random | “  “So just two days ago I had an Autopilot software crash.‬ I’m not entirely sure what happened, but it does occasionally happen during this test process. I’ve never seen FSD Beta crash.” (R094)‬‬‬‬‬‬‬‬‬‬‬‬‬‬‬‬‬‬‬‬‬‬‬‬‬‬‬‬‬‬‬‬‬‬‬‬‬‬‬‬‬‬‬‬‬‬‬‬‬‬‬‬‬‬‬‬‬‬‬ |
| False positives | False positive, false positives | “There’s been a time where I was wearing sunglasses, and it couldn’t be sure that I was actually paying attention, and so it gave one of the red steering wheel warnings to take over, and that’s essentially the biggest times that it’ll disengage if it thinks that you’re not paying attention.” (R037)  “If I have to get yelled at by the camera all the time because it’s literally tracking my eyeballs, and it’s wrong sometimes. If it sees a phone, it has really good AI, even if you’re looking at it, a map or whatever, it just sees the device in your hand, and then you get a strike, right? So, I have two strikes out of five already, and I didn’t buy a car to get a strike out.” (R042) |
| Harsh deceleration | Harsh deceleration, phantom, phantom braking, braking, rear end, rear-end | “I was encountering a lot of phantom braking. There were elements of safety involved. If I’m on the highway, my car just decides to randomly brake hard. If there’s someone behind me, that might be an accident. So, I just stopped using it unless there was no one around me. ‘Nope. OK, I’m out for a while. I don’t need that again.’” (R056)  “When it does that phantom braking, then it makes me feel anxious, and I immediately leave Autopilot.” (R059) |
| Erratic steering wheel movements | Erratic, jerk, jerks, jerked, jitters, jittering, jerky | “You don’t ever know what it’s gonna do. Jerks the wheel back and forth. ‘What are you doing? This is not what we do.’ So, you slam on the brake, and you take over and drive.” (R084)  “The jerkiness of it sometimes makes it feel it may randomly ram another car. Typically, those are things where I would end up taking over because I don’t trust it.” (R090) |
| Steering into adjacent traffic | Oncoming, oncoming traffic, oncoming lane, oncoming lanes | “It will take the outside lane until the last second, and it will cut over into the inside lane, which can be an accident, and so you have to babysit it. It’s not a feature yet. It will turn into traffic if you let it.” (R011)  “It drove into the wrong lane. It got confused, and then actually drove into the oncoming traffic lane. I’ve had that happen.” (R042) |
| Route unfamiliarity | Unfamiliar, unknown, not familiar, new area, new city | “Full-Self-Driving Beta ‒ sometimes when I’m in an area where I’m not familiar with, and that’s when I would disengage it.” (R020)  “FSD Beta ‒ in areas that I drive common, I use it almost all the time. If I were to go into a new city, I most likely won’t use it. Maybe if I’m there for a week, the first few days I won’t use it just because I want to see what the area and roads are like.” (R075) |
| Taking the wrong route | Navigation, mapping, location | “It can be driving along, and then be like ‘I’m gonna turn left.’ Turn left with no blinker or anything. So, let’s say it was supposed to turn left, but it didn’t, and it continued straight, and then the navigation re-navigates, and just suddenly turned left across multiple lanes.” (R002)  “There is one specific corner on the freeway where the map data is wrong, so I never use Autopilot there. I’ll switch it off, drive around this corner, and turn it back on.” (R038) |
| Undesired lane changes | Change lanes, changes lanes, lane change, lanes changes | “So, it was like ‘I’ll change lanes’, and I’ll be thinking ‘Why the heck are doing that for?’ ‘I must say, I’m gonna right lane, I’m gonna make a right turn.’ It’ll go to the left lane for something, and then go back to the right lane again, and I’m thinking ‘You know why I’m staying in the right lane?’ You needed game theory going on in the left lane. Just turn it off because it will do things that maybe wasn’t dangerous, but it just wasn’t necessary. (R072)  “It’s trying to make a lane change and it’s just freaking the other drivers out. Sometimes it’ll try to make a lane change on the freeway, and there won’t be quite enough space between people, so it’ll just stay there, and stick there with blinker on, either holding up traffic or confusing the people behind me so then I feel unsafe, but then I just take control of the car.” (R098) |
| Misidentifying correct lane | Parking lane, bus lane, bike lane, incorrect lane,  incorrect lanes | “It will almost always choose the wrong lane to be in. Sometimes it’ll get in the parking lane. Sometimes it’ll get in the correct lane. You never know. So, you really have to pay attention to that.” (R010)  “It does some weird things when it’s coming across a bike lane. It mainly let me try going to the bike lane. Those things get better over time as people report them.” (R034) |
| Creeping | Creep, creeps, creeping | “If you make a right turn, and you need to get over multiple lanes immediately, the car will always do a right turn, and then take its sweet time to get over, and usually it seems it’s going to be stuck getting, so I will just do it myself.” (R050)  “FSD Beta, I manually have to override it to make it go through an intersection faster, or else I’ll miss my chance, and then confuse other people in intersections.” (R060) |
| Rolling through  stop signs | Rolling through stops, rolling stops | “When it comes up to a stop sign, it’ll be so slow stopping. If there’s someone behind me or other cars around, I’ll usually turn it off because it’ll be so slow at making decisions. It’ll confuse everybody. So, I’ll take over a lot of times.” (R021)  “At an intersection, a lot of times I’ll just take over because it’s too awkward if it hesitates too long to go. I don’t let Autopilot sit there.” (R096) |
| Running red lights / stop signs | Running red, running stop, run red, ran red, run stop, ran stop | “FSD basically tries to run this red light. This is illegal. Illegal right turn. I stopped because it can’t see beyond that truck. There’s no way that it knows it has the right of way.” (R011)  “It can run a stop sign. If the stop sign is not on the map to let the car know you have to stop here, the vision system cannot see and recognize with enough distance to give it enough time to come to a stop. I tested yesterday at 45, and in fact it ran the stop sign and then came to a stop afterwards on the other side.” (R096) |
| Unprotected left turn | Unprotected, unprotected left, unprotected left turn, unprotected left turns, unprotected right unprotected right turn, unprotected right turns | “It’s an unprotected left turn, and the car will creep forward at the wrong time, when a car is just crossing, and that can be really scary. ‘Oh, is the car just gonna run into this car? Why is it moving forward if there’s traffic?’” (R027)  “It was waiting to make a left-hand turn, and it started jittering, and moving forward a little bit when there was clearly traffic. Full Self-Driving realized ‘OK, I can move a little bit forward, I can move a little bit forward, and maybe there’s a gap here.’ There’s no gap there. I wasn’t willing to take that risk. It will probably crash the car.” (R045) |
| Other turning situations | Turn, turns, protected left, protected right, protected left turn, protected left turns, protected right turn, protected right turns, turning, turn on red, turning on red | “A typical example is drive up to a four-way-stop signs. Sometimes you have cars around. We need to figure out who takes the turn, and FSD Beta doesn’t actually do a very good job of going up at the full stop sign. It’ll always wait for other cars, and so usually I like to press the accelerator pedal to kind of get to go.” (R003)  “At a traffic light, it was gonna make a left turn. It was red, and green, cars were coming, and the wheel was moving. It was hesitating, thinking, and it was about to go. Two lanes of cars coming towards me. So, I had to jam on the brakes, and I hit the capture button like ‘What on earth are you doing? You see everything else. You can’t see these two lines of cars coming at you?’ That would have been an accident.” (R052) |
| Discomfort | Passenger, passengers, wife, discomfort, lack of control, unpredictable, unpredictability | “I know people who say ‘Hey, I’m not driving Autopilot with my kids in the car’, and my wife says, ‘You’re not using that thing with the kids in the car.” (R008)  “My wife will not accept the FSD Beta being turned on because she does not trust it mostly because the way that it reacts to things can be very jerky and not very smooth. So, it’s the harshness, and the non-warning of those situations that makes her very uncomfortable as a passenger and she won’t use it herself when she’s driving the car.” (R090) |
| Road rage | Rage, road rage, angry, annoyed, confusion, confused | “Drivers behind you are going to get annoyed and frustrated and that might cause them to behave in an anxious or unsafe way. That would put me in an unsafe condition, so I would intervene.” (R012)  “I really don’t have an intervention unless somebody’s behind me pushing me, and they get frustrated because it takes a turn too slow for them, and then I’ll just disengage.” (R062) |
| Reckless behavior | Reckless, follow too close, followed too close, following too close, swerve, swerved, swerving, cut, cutted, cutting | “Somebody coming up behind you too quickly is they don’t expect the car to be there. That there are people driving around you way too aggressively who might be impatient, not wait for the car.” (R065)  “I typically don’t have to intervene most of the trip unless it’s a situation that’s more caused by another driver driving erratically or there’s something along those lines that I don’t trust the system enough.” (R090) |
| Complacency | Complacency, complacent, monitor, not monitor, jail | “So, if you’re paying attention, it’s no problem. If you’re not, that thing starts panicking. All it really does is basically slow down, and slam on the brakes, and stopped.” (R006)  “Autopilot both disengage in some scenarios if you don’t pay attention to the road a certain number of times. Autopilot will make itself not available if you’ve reached that number of strikes for the remainder of that drive.” (R031) |
| Torque | Torque, accidentally | “It doesn’t take much to disengage it. I might hover my hand over the wheel. That way, I don’t accidentally come disengage it during a turn.” (R032)  “So, what happens is on a curve, it turns the steering wheel. So, your hands are on it, but if it wants you to nudge it on a turn, there’s a chance you nudge it a little too much, and then you’re on a turn with no Autopilot anymore.” (R070) |
| Speed rule violation | Exceed speed, exceeded speed, speed limit, above speed, over speed | “Sometimes you’ll go over the limit, and the car will start to scream at you. I have to manually turn it off. I can speed around people, and then I can turn it back on.” (R056)  “When I first bought the car there was nobody else around. I simply went too fast, and Autopilot kicked me off because I noticed that I went above 90 mph‬.” (R066) |
| Weather | Inclement weather conditions, inclement weather, bad weather, poor visibility, rain, fog, snow, slug | “It did disengage one other time. There was like a slush on the freeway, so Autopilot wouldn’t work because all the slush was covering the radar.” (R043)  “Most of the times that it would disengage would be weather-related.‬ There’s certain times where you’re getting rain or snow, and it detects that you can’t engage FSD Beta, it’ll tell you there’s inclement weather, you can’t use it.‬” (R087)‬‬‬‬‬‬‬‬‬‬‬‬‬‬‬‬‬‬‬‬‬‬‬‬‬‬‬‬‬‬‬‬‬‬‬‬‬‬‬‬‬‬‬‬‬‬‬‬‬‬‬‬‬‬‬‬‬‬‬‬ |
| Non-standard roads | Inconsistent lane, inconsistent lanes, marking, markings, road, roads, width | “If there’s an exit lane with no dotted line, the car thinks the lane is very wide, and it’s always trying to center itself between what it thinks is the lane, so it might center itself between the old lane and the new exit lane, and then it comes to the separation point, and makes some last second crazy decision. I’ll just turn the system off.” (R007)  “It doesn’t really know how to deal with unmarked roads well yet. It will try to stay in either the center of the road, and then it’ll go to the right of the road, and then it’ll go very slowly because it doesn’t know if the edge of the road is really the edge of the road. So, I’ll usually have to take over for that situation.” (R074) |
| Curves, hills | Tight turn, tight turns, curve, curves, hill, hills | “There is one specific scenario that was so scary. It’ll get into the turn lane, and then it suddenly lurches to the right. It ends up leaving the lane, and it will turn, and if you have not turned it back, it would have crashed into something, right?’” (R011)  “It struggles a little bit with tight turns and creeping. There’s a balance, you want it to learn, but you don’t wanna annoy people and you don’t want to have to take over if you don’t have to.” (R100) |
| Objects and events | Pothole, potholes, curb, curbs, barrier, barriers, object, objects, cone, cones, debris, road debris, bump, bumps, bush, bushes, tree, trees, building, buildings, parked, stopped, construction, constructions, crossing, crossings, railway crossing, railway crossings, gate, gates, VRU, VRUs, vulnerable road user, vulnerable road users, road user, road users, pedestrian, pedestrians, motorcyclist, motorcyclists, cyclist, cyclists, emergency vehicle, emergency vehicles, animal, animals | “I’ve had moments where Beta immediately started making a sharp left into a tree by the side of my driveway, and if I had not intervened, it probably would have hit the tree.” (R068)  “I do not trust it around pedestrians and small children and dog walkers because it’s too close to a human. That Tesla will approach the pedestrian and giving them a very small birth, and they would be like ‘What are you doing? There’s plenty of room.’ It’s not quite human so I don’t always trust it around humans because it’s not as courteous as a human would be, right?’” (R031) |
| Intersections | Intersection, intersections, roundabout, roundabouts | “When you’re at an intersection, and there’s four cars at this intersection, and they’re all trying to encourage the other driver to go first. The car doesn’t really recognize that. I will have to either take over, or I’ll have to hope that the car goes pretty soon.” (R017)  “Especially roundabouts. It is just far too unsure.‬ It’s very jerky at times, it’ll stop and start, stop, and start, and when you’ve got other traffic around you, I don’t wanna deal with that. So, I’ll typically turn it off. I typically only use it for highway driving.‬” (R056) |
| Discontinuities in road design | Ramp, ramps, on-ramps, off-ramps, merging, splitting, exit | “I’ve had it disengaged near off ramps where it just doesn’t understand that the lane is diverting.‬” (R028)‬‬‬‬‬‬‬‬‬‬‬‬‬‬‬‬‬‬‬‬‬‬‬‬‬‬‬‬‬‬‬‬‬‬‬‬‬‬‬‬‬‬‬‬‬‬‬‬‬‬‬‬‬‬‬‬‬‬‬‬‬‬‬‬‬‬‬ |
| Complex, heavy traffic | Traffic, heavy traffic, complex traffic, other cars, many cars | “So, if it was two in the morning, and there was no one else on the freeway, I let it do its thing and not disengage, but usually there would be traffic around, so usually I’d need to disengage.” (R007)  “I use it in areas where I would feel comfortable training a brand-new driver, and so I don’t feel safe using it on busy streets.” (R059) |
