# Supplementary material for: A conceptual framework for automation disengagements
Source: Sci Rep. 2024 Apr 15;14:8654. doi: 10.1038/s41598-024-57882-6 (PMC11018869; doi:10.1038/s41598-024-57882-6)
Supplement: Supplementary file 2 — Supplementary Information 2. [file 41598_2024_57882_MOESM2_ESM.pdf]

```python
# import pandas as pd
import os
from docx import Document
import re
from nltk.tokenize import sent_tokenize, word_tokenize

# Create an empty DataFrame with columns 'participant' and 'conversation'
data_training = pd.DataFrame(columns=['participant', 'conversation'])

# Assuming all Word files are in the same directory as your Python script
transcript_directory =
'/System/Volumes/Data/Users/snordhoff/Desktop/Kursmaterialien/01 - Python
Bascis'

# Initialize a dictionary to store the data
transcript_data = {}

# Function to replace newline characters
def remove_newline(text):
    # Replace newline characters with an empty string
    text_without_newlines = text.replace('\n', ' ')

    return text_without_newlines

def add_space_between_number_and_alphabet(text):
    # Use a regular expression to match a digit followed by an alphabet
character
    # and replace it with a space-separated version
    modified_text = re.sub(r'(\d)([a-zA-Z])', r'\1 \2', text)
    return modified_text

def preprocess_text(text):
    text_without_newlines = remove_newline(text)
    text_without_space =
add_space_between_number_and_alphabet(text_without_newlines)

    return text_without_space

# Loop through all Word files in the directory
for filename in os.listdir(transcript_directory):
    if filename.endswith('.docx'):
        doc_path = os.path.join(transcript_directory, filename)
        doc = Document(doc_path)
        content = " ".join([para.text for para in doc.paragraphs])

        # Preprocess content
        conversation = preprocess_text(content)

        # Extract participant name from filename
        participant = filename[:-5]

        # Create a DataFrame for the current data
```

```python
        df = pd.DataFrame({'participant': [participant], 'conversation':
[conversation]})

        # Concatenate the current DataFrame with the main DataFrame
        data_training = pd.concat([data_training, df], ignore_index=True)

# Sort the DataFrame by 'conversation'
data_training = data_training.sort_values(by='conversation')
data_training

# Tokenize the 'conversation' column into sentences
data_training['sentences'] =
data_training['conversation'].apply(word_tokenize)

# Sort the DataFrame by 'conversation'
data_training = data_training.sort_values(by='conversation')
data_training
# Count number of tokenized sentences
num_tokenized_sentences = data_training['sentences'].apply(len).sum()
print(f"Total number of tokenized sentences: {num_tokenized_sentences}")
# Clean conversation
from collections import Counter
import pandas as pd
import nltk
from nltk.corpus import stopwords
from nltk.tokenize import word_tokenize, sent_tokenize
from nltk.stem import WordNetLemmatizer
import re
from gensim import corpora

def clean_text(tokens):
    # Remove words with less than two letters
    tokens = [token for token in tokens if len(token) > 2]

    # Remove spurious characters and words longer than 30 letters
    tokens = [token for token in tokens if len(token) <= 30 and
token.isalpha()]

    # Remove numbers and punctuation
    tokens = [token for token in tokens if not token.isdigit()]

    # Transform letters to lowercase
    tokens = [token.lower() for token in tokens]

    # Remove stopwords
    stop_words = set(stopwords.words('english'))
    tokens = [token for token in tokens if token not in stop_words]

    # Remove specific words
    words_to_remove = ['autopilot', 'nordhoff', 'thankful','uh', 'plugging',
'app', 'autohotkey', 'echoing', 'session', 'motivate', 'ftp', 'lie',
'stuff','adds', 'matrix', 'et', 'items', 'questionnaire', 'added', 'cetera',
'square', 'played', 'issues','ultimate', 'wrapped', 'administration',
```

```
'enhances', 'people', 'impinge', 'promotes', 'layout', 'seeds', 'realm',
'part', 'wan','comes','last','self', 'coming','come', 'tell',  'getting',
'got','questions', 'new',
'may','done','another','course','without','give','different','first',
'said', 'thank', 'trying', 'feel', 'see', 'used', 'every', 'might',
'looking', 'many', 'done', 'another', 'try', 'able', 'driving', 'use',
'take', 'things', 'using', 'two', 'put', 'makes', 'fsd', 'beta', 'got',
'tesla', 'anything', 'nothing', 'think', 'sometim', 'get',
'gave','say','make', 'link', 'make', 'car','could', 'always', 'even', 'also',
'around', 'one', 'around', 'everything', 'really', 'something', 'next',
'whatever', 'sorry', 'zoom', 'much', 'quit', 'oh', 'guess', 'gonna', 'yes',
'would', 'av', 'na', 'sure', 'general', 'san','question', 'great', 'fine',
'actually', 'interesting', 'little', 'let', 'nice', 'bit', 'thing',
'answer','francisco', 'kind', 'mean', 'overall', 'maybe', 'umm', 'way',
'thing', 'need', 'Sina', 'lot', 'question', 'uhm', 'example', 'want',
'going', 'yeah', 'like', 'right', 'already', 'Nordhoff', 'sina', 'exactly',
'know', 'thanks', 'ok', 'actually']
    tokens = [token for token in tokens if token not in words_to_remove]

    return tokens

tokens = data_training['sentences'].apply(clean_text)
tokens

data_training['sentences']
45    [00:00:00.000, --, >, 00:00:03.830, Sina, Nord...
30    [And, we, can, discuss, it, and, yeah, ,, OK, ...
89    [00:00:00.000, --, >, 00:00:00.260, Sina, Nord...
5     [00:00:00.000, --, >, 00:00:00.260, Sina, Nord...
9     [00:00:00.000, --, >, 00:00:00.390, Sina, Nord...
                              ...
16    [Sina, Nordhoff, Hi, ., 01:18:18.060, --, >, 0...
48    [Sina, Nordhoff, Hi, ., Hey, ,, am, I, an, hou...
96    [Uh, ,, yeah, ,, so, I, definitely, watched, q...
69    [We, do, have, the, full, side, driving, featu...
92    [Yes, ,, which, we, were, ,, uh, ,, we, could,...
Name: sentences, Length: 104, dtype: object
# Define seeded topics
seeded_topics = [
["intervene" , "traffic"]

    # Safer than human drivers
    # Add more seeded topics as needed
]

# Convert dictionary values into a list
keywords =["intervene" , "traffic"]

n_occurrences_threshold = len(keywords) # Minimum unique keyword occurrences
in the buffer

# Initialize relevant content list
relevant_content_list = []
```

```
# Initialize document index
document_index = []

# Step 2 and 3: Matching Keywords and Buffered Context Extraction
buffer_size = 10  # Number of tokens in the buffer

for i, keywords in enumerate(seeded_topics):

    for k in range(0, len(tokens)):
        relevant_content = []
        for i, token in enumerate(tokens[k]):
            if token in keywords:
                start_index = max(0, i - buffer_size)
                end_index = min(len(tokens[k]), i + buffer_size + 1)
                context = tokens[k][start_index:end_index]
                relevant_content.append(' '.join(context))
        relevant_content_list.append(relevant_content)


    final_relevant_content_list = []

    for k in range(0, len(tokens)):
        final_relevant_content = []
        relevant_content = relevant_content_list[k]
        for context in relevant_content:
            unique_keyword_count = len(set(keyword for keyword in keywords if
keyword in context))
            if unique_keyword_count >= n_occurrences_threshold:
                document_index.append(k)
                final_relevant_content.append(context)
        final_relevant_content_list.append(final_relevant_content)

    non_empty_count = sum(1 for sublist in final_relevant_content_list if
sublist)
    print('Seeds: ', keywords)
    print(f"Number of occurences: {non_empty_count}")
    print('')

    doc_index = list(set(document_index))
    doc_index.sort()

    for num in doc_index:
        print('Document title: ', data_training.iloc[num, 0] + '.docx')
        print('Selected tokens: ')
        for content in final_relevant_content_list[num]:
            print('- ' + content)
        print('')


print('=============================================================================
==============')
```
